# Functional characterization of a unique cytochrome P450 in *Toxoplasma gondii*

**DOI:** 10.18632/oncotarget.23023

**Published:** 2017-12-06

**Authors:** Xiao Zhang, Taotao Zhang, Jing Liu, Muzi Li, Yong Fu, Jianhai Xu, Qun Liu

**Affiliations:** ^1^ Key Laboratory of Animal Epidemiology and Zoonosis, Ministry of Agriculture, College of Veterinary Medicine, China Agricultural University, Beijing, China; ^2^ National Animal Protozoa Laboratory, College of Veterinary Medicine, China Agricultural University, Beijing, China

**Keywords:** T. gondii, P450, biotransformation enzymes, drug resistance

## Abstract

The basic metabolic cytochrome P450 (CYP) proteins are essential for the biotransformation of sterols and xenobiotics. By contrast, the *Toxoplasma gondii* genome contains only one CYP gene, and the role of this enzyme in the physiology and biochemistry of apicomplexan parasites is unknown. Because it is a potential resistance gene, identifying the functionality of P450 in *T. gondii* is particularly important. Knocking out *Tg-P450* had no significant effect on *T. gondii* survival, but mice infected with parasites overexpressing *Tg-P450* exhibited significantly enhanced pathogenicity. Enzyme activity analyses demonstrated that this protein has mammalian CYP2B and CYP3A enzymatic activity. In addition, *T. gondii* lacking the P450 gene exhibited reduced resistance to quinine, mefloquine and clarithromycin compared with parasites overexpressing Tg-P450. These results suggest that P450 functions in *T. gondii* metabolism and detoxification is involved in vitally important processes in parasitic organisms, making this enzyme a potential drug target.

## INTRODUCTION

*T. gondii* is a globally ubiquitous pathogen that infects approximately 30% of the world population. This obligate intracellular parasite is found in virtually all warm-blooded vertebrates, with feline species serving as the definitive host [1]. As a major opportunistic infection protozoan parasite, toxoplasmosis is usually reported in low-immunity populations, such as developing countries wherein HIV/AIDS is rampant, and it commonly causes focal brain lesions, coma and death [2]. The absence of an effective vaccine against *T. gondii* infection and its growing drug resistance makes it more important to understand the physiology of *T. gondii* and explore new drug targets. Importantly, *T. gondii* can resist the killing effects of many drugs that have previously proven to be effective against *Plasmodium falciparum*, also an apicomplexan [3]. We hypothesize that some drug resistance genes exist in *T. gondii* that are associated with this phenomenon.

Heme-containing cytochrome P450 enzymes (CYPs, also known as CYP450s) are widely distributed in living organisms, ranging from microorganisms to mammals. Their ubiquity may reflect their ancient origin and physiological importance. CYPs mainly catalyze monooxygenase reactions, metabolizing a wide variety of endogenous substances, such as steroids and fatty acids [4, 5] and detoxifying exogenous substrates. Thus, elevated CYP activity accelerates drug metabolism and is often a reason for drug resistance. While the human genome encodes 57 CYPs, one enzyme, CYP3A4, catalyzes more than 50% of these reactions [6, 7]. Despite their presence in all phyla of living organisms, some parasites are long thought to lose both CYPs and their ability to oxidize xenobiotics [8]. Nonetheless, CYP-like activity was later identified in some apicomplexan parasites (*Plasmodium falciparum* [9] and *Trypanosoma cruzi* [10]), and the role of this activity in parasite drug-resistance was demonstrated [8, 11] by proving the existence of the CYP system. However, no data on the composition of the P450 system or the structure and expression of these proteins in *T. gondii* are available.

The aims of this study were (i) to determine the significance of P450 for *T. gondii* survival and pathogenicity, (ii) assess the role of P450 in drug resistance, (iii) and reveal an essential xenobiotic biotransformation mechanism for the survival of *T. gondii* lacking Tg-P450.

## RESULTS

### Detection of only one CYP450 protein in *T. gondii*

**Figure 1:**
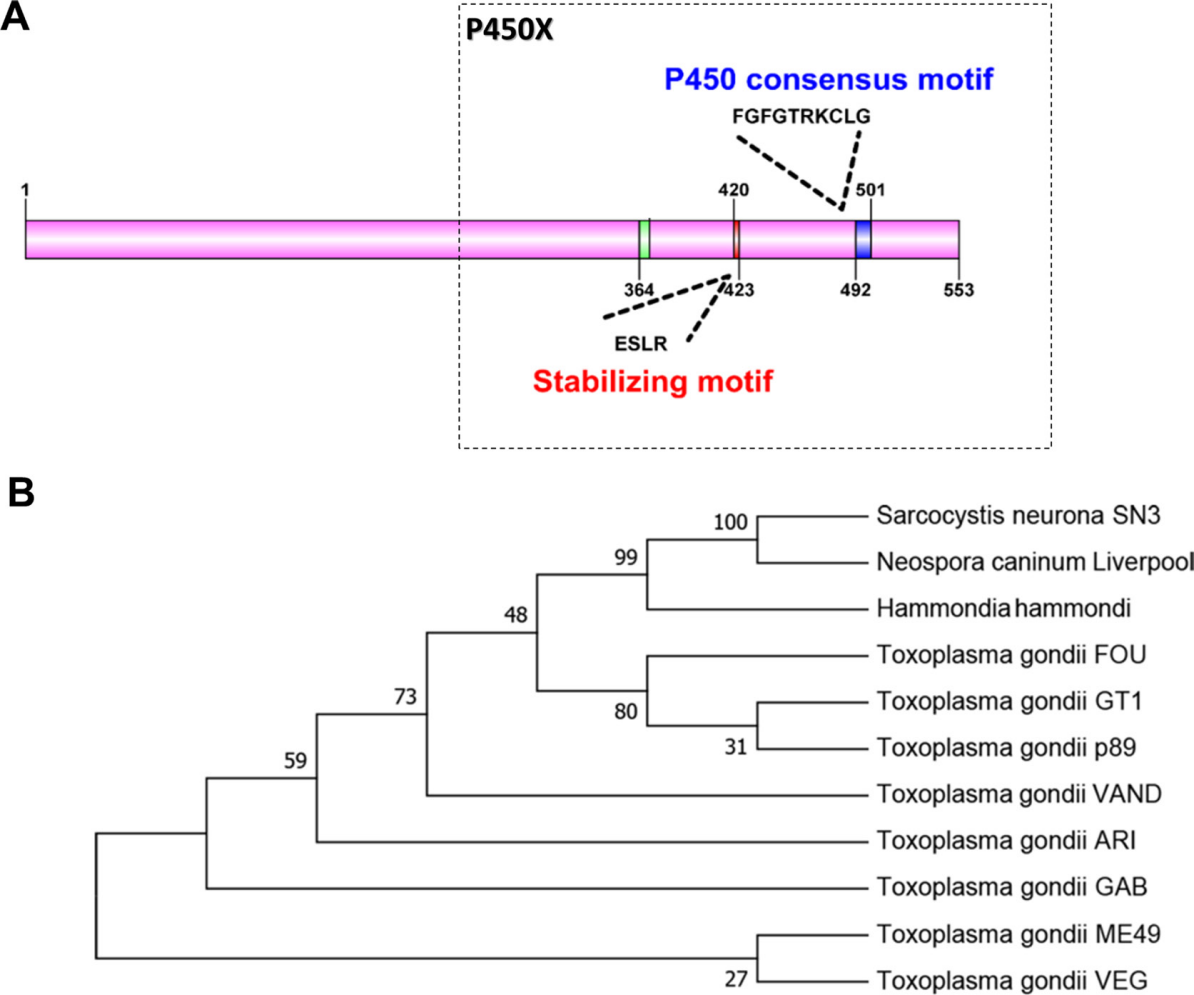
Comparison of the Tg-P450 protein with CYP450 proteins from other species (**A**) Protein sequence-based schematic representation of conserved CYP450 motifs in the Tg-P450 sequence. (**B**) Phylogenetic analysis of Tg-450 with Neospora caninum, *Hammondia hammondi*, *Sarcocystis neurona* and multiple *T. gondii* strains annotated in the ToxoDB database generated using the Neighbor-Joining method. The bootstrap consensus tree inferred from 1000 replicates is taken to represent the evolutionary history of the taxa analyzed. Branches corresponding to partitions reproduced in less than 50% bootstrap replicates are collapsed. The percentages of replicate trees in which the associated taxa clustered together in the bootstrap test (1000 replicates) are shown next to the branches.

Only one P450 gene (TGGT1_315770) was found among all the *T. gondii* genome sequences annotated in the ToxoDB database. Analysis of the Tg-P450 coding region sequence and a search for conserved domains using the National Center for Biotechnology Information (NCBI) conserved domain database revealed the presence of a functional domain (*E*-value = 3.85e-31) belonging to CypX, a member of the cl12078 superfamily (https://www.ncbi.nlm.nih.gov/Structure/cdd/wrpsb.cgi) that plays roles in catabolism and defense. Therefore, we believe that Tg-P450 may have a vital effect on metabolic exogenous drugs. The Tg-P450 C-terminal region contains a heme-binding loop with a characteristic P450 consensus motif (Phe-X-X-Gly-X-Arg-X-Cys-X-Gly), with this specific motif comprising Phe^492^-Gly-Phe-Gly-Thr-Arg-Lys-Cys-Leu-Gly^501^ (Figure 1A), which helps position the iron atom in the heme [12, 13]. Another conserved region is the Glu-X-X-Arg motif in helix K, which usually helps stabilize the protein core. Other conserved structural regions also exist in Tg-P450, such as the PERF motif Pro^476^-Asp-Arg-Phe^479^ and the helix I motif Lys^441^-Ser-Val-Asp-Phe-Ser^446^.

Sequence analysis was performed using P450 protein sequences from *Neospora*, *Hammondia* and *Sarcocystis*, which are similar to *T. gondii*. Tg-P450 shares 96% sequence identity with the *Hammondia* protein, 79% sequence identity with the *Neospora* protein and 56% sequence identity with the *Sarcocystis* protein. Phylogenetic analysis showed P450 to be relatively conserved in the different strains of *T. gondii* (Figure 1B). These results indicate that P450 may have similar functions in *T. gondii*.

### Tg-P450 is unnecessary for the survival of *T. gondii* tachyzoites

**Figure 2:**
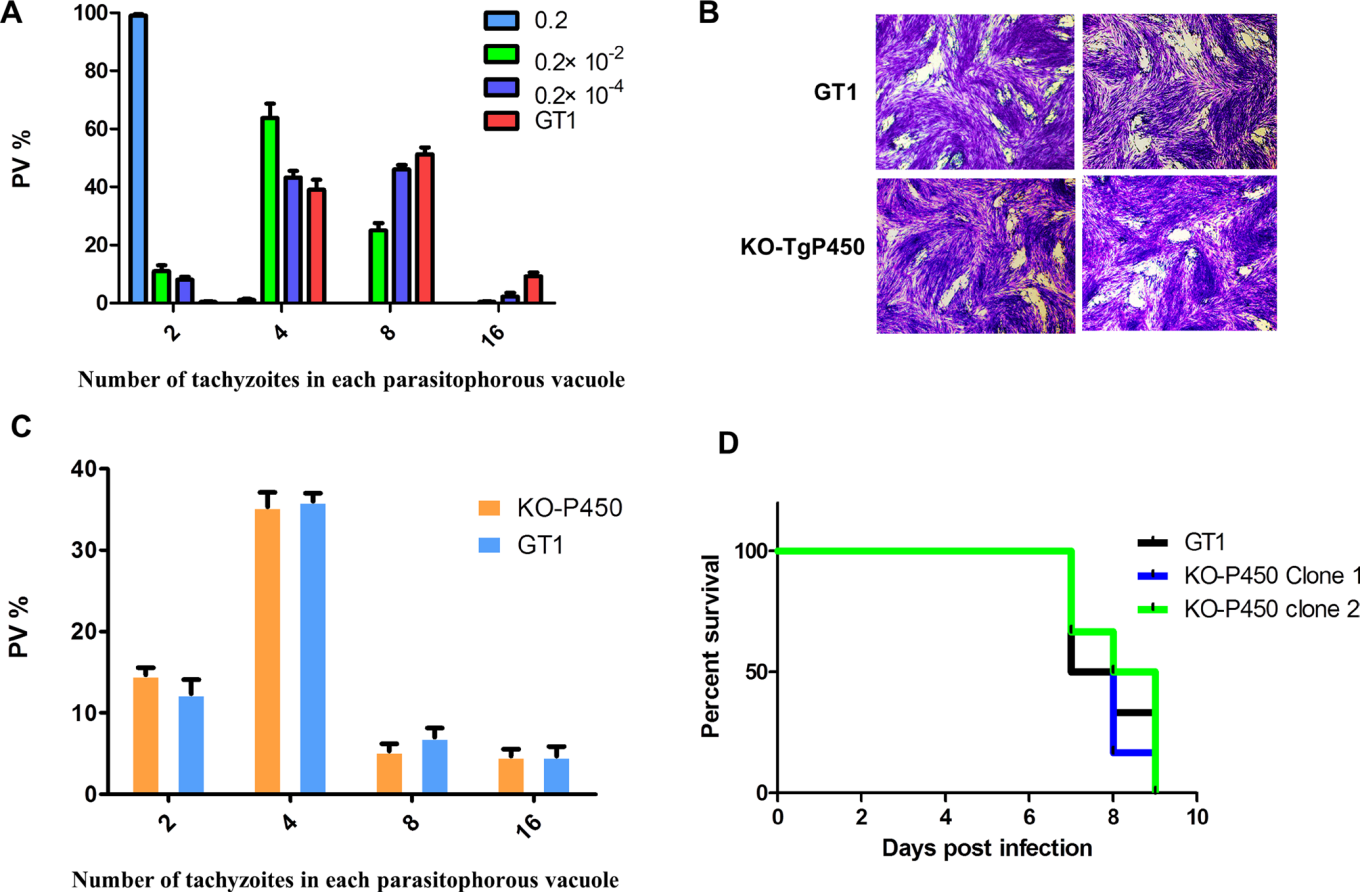
Knocking out Tg-P450 has no effect on *T. gondii* proliferation (**A**) Proliferation of tachyzoites per 100 host cells in cultures treated with KCZ at concentrations of 0.2 mol/L, 0.2 × 10^−2^ mol/L, and 0.2 × 10^−4^ mol/L. The number of tachyzoites per cell was assessed by IFA. Statistical information is in Supplementary Table 1. Note: Comparisons were made with the control group (GT1). (**B**) Plaque assay comparing the viabilities of the KO-Tg-P450 and GT1 strains. (**C**) Proliferation of KO-Tg-P450 tachyzoites per 100 host cells compared with that of GT1 tachyzoites in 24 h. (**D**) Mouse death curve. Pathogenicity in mice infected with KO-Tg-P450 strain tachyzoites compared with that of mice infected with GT1 strain tachyzoites; 100 tachyzoites/mice. Six mice were assigned to each group, and the statistical results are shown above.

In other organisms, P450 proteins have been shown to be vital for survival. After treatment with ketoconazole (KCZ, an inhibitor of CYP3A4) at final concentrations of 0.2 mol/L, 0.2×10^−2^ mol/L, and 0.2×10^−4^ mol/L, the proliferative capacities of the parasites were significantly decreased. As the KCZ concentration increased, the inhibitory effect on the proliferation of *T. gondii* became stronger (Figure 2A). Therefore, we believe that Tg-P450 is essential for *T. gondii* growth. To further verify the function of Tg-P450, we used CRISPR-Cas9 gene deletion techniques to create *Toxoplasma* tachyzoite lines with Tg-P450 knocked out. The knockouts were confirmed by Western blot analysis with anti-P450 antisera (Prepared by our laboratory) (Supplementary Figure 1D). To assess the survivability of the Tg-P450 knockout parasite line, an integrative plaque assay was performed to assess a multi-step process involving invasion, several rounds of replication and egress. When parasites were cultured for 7 days, no plaque difference was observed between the KO-Tg-450 strain and the parent GT1 strain (Figure 2B). Proliferation experiments also showed no significant difference between the KO-Tg-P450 strain and the GT1 strain within 24 hours of culture (Figure 2C). Thus, these results revealed that P450 is unnecessary for *T. gondii* tachyzoite survival, at least under normal culture conditions. Subsequent mouse survival experiments showed that KO-Tg-P450 slightly reduced the pathogenicity of *T. gondii* in mice, but the reduction was not statistically obvious (Figure 2D).

### Tg-P450 overexpression increases susceptibility to *T. gondii* infection in mice

**Figure 3:**
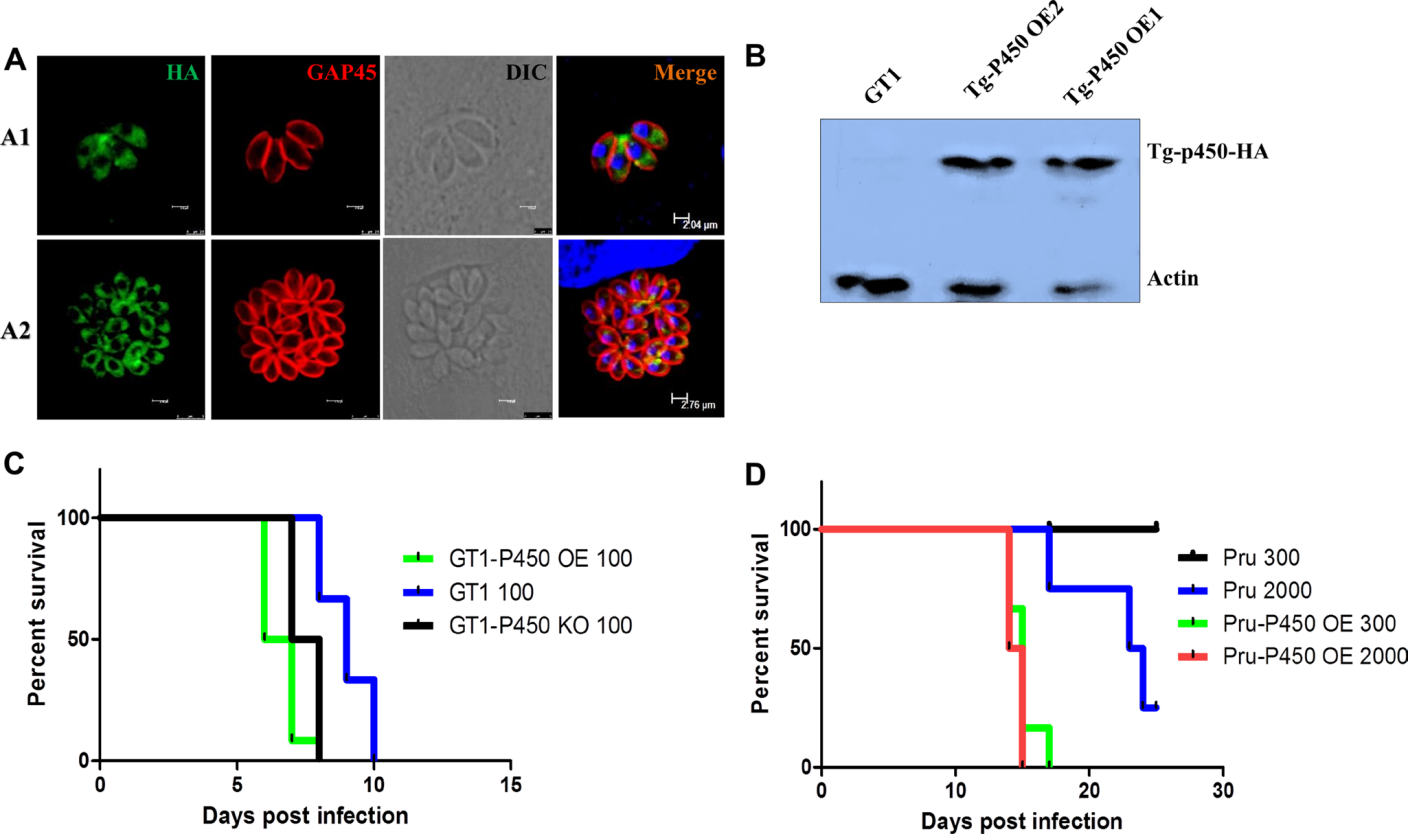
Overexpressing Tg-P450 enhances the pathogenicity of *T. gondii* (**A**) IFA showing Tg-P450-HA expression levels. Green indicates Tg-P450 detected with an anti-HA antibody (Sigma, H3663, USA), and blue indicates cell nuclei. TgGAP45 was stained red to observe the shape of the parasites. Note: A1, cultured for 12 hours; A2, cultured for 48 hours. (**B**) Western blot analysis of Tg-P450-HA expression levels. The hemagglutinin (HA) label protein was fused with the target protein, and expression was detected. (**C**) Mouse death curve. Pathogenicity in mice infected with Tg-P450 OE (GT1) strain tachyzoites compared with that of mice infected with GT1 and KO-Tg-P450 strain tachyzoites; 100 tachyzoites/mice. Six mice were assigned to each group, and the statistical analyses are shown above. (**D**) Mouse death curve. Pathogenicity in mice infected with Tg-P450 OE (Pru) strain tachyzoites compared with mice infected with Pru strain tachyzoites; 300 or 2000 tachyzoites/mice. Six mice were assigned to each group, and the statistical analyses are shown above.

To further verify the function of Tg-P450, we also constructed a Tg-P450 overexpression strain (Tg-P450 OE), which was electroporated with modified pDMG-TgMCA into GT1, which is a type I strain. After several rounds of selection by pyrimethamine, a transgenic GT1 strain stably expressing Tg-P450 fused with a hemagglutinin (HA) tag was isolated and confirmed by Western blot (Figure 3B). An IFA showed that the Tg-P450 protein was expressed in the *T. gondii* cytoplasm (Figure 3A). The pathogenicity of the Tg-P450 OE strain was measured in BLAC/c mice. All mice infected with the Tg-P450 OE strain died within 7 days, but GT1 tachyzoite-infected mice survived three days longer (Figure 3C). Tg-P450 OE Pru, a type II strain, was also constructed and used to verify the pathogenicity variation. Virulence lethality in mice inoculated with 2000 and 300 Tg-P450 OE Pru tachyzoites, while a lower pathogenicity was found in mice inoculated with the Pru parent (Figure 3D). These results suggest that Tg-P450 may play special roles in helping *T. gondii* resist factors that are not suitable for survival.

### *T. gondii* has CYP enzyme activities

**Figure 4:**
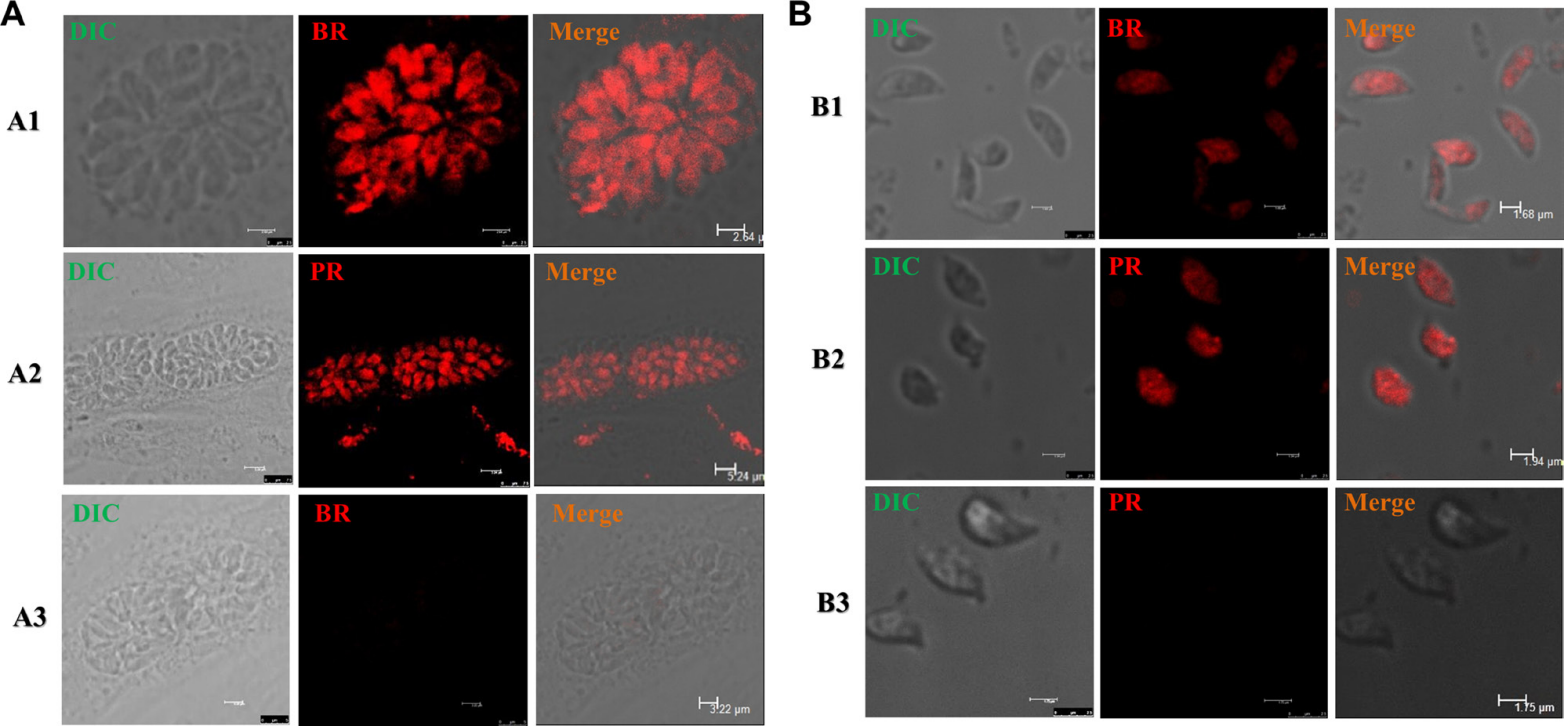
*T. gondii* CYP activities *in situ* Fluorescence micrographs of *T. gondii* tachyzoites exposed with several substances for 24 h. (**A**) Intracellular tachyzoites were treated with benzoxyresorufin (BR) and pentoxyresorufin (PR). Note: A1-A2, GT1 strain; A3, KO-Tg-P450 strain. (**B**) Extracellular tachyzoites were treated with benzoxyresorufin and penzoxyresorufin (rhodamine). Monochrome images were acquired using a rhodamine filter. Note: B1-B2, GT1 strain; B3, KO-Tg-P450 strain.

PR and BR are fluorogenic substrates of cytochrome P450, and a fluorescent product (resorufin) is produced upon enzymatic cleavage of the alkyl group [14]. If *T. gondii* does in fact possess monooxygenase ability, the parasites will produce a fluorescent product observable under a rhodamine filter. After incubation with PR and BR for 24 h, a weak specific fluorescence signal was visible in the Tg-P450 OE strain (Figure 4A1, A2, B1, B2) but not in the host cells (HepG2). After treating the P450 knockout parasites with PR and BR, no fluorescence was observed (Figure 4A3, B3). Therefore, *T. gondii* can metabolize substrates specific to monooxygenases of the mammalian CYP2B and CYP3A families (PR and BR, respectively) [15]. Unfortunately, we did not clearly observe the location of metabolism because of the weak fluorescence, which also indicated that the CYP enzymatic activity in *T. gondii* is extremely weak.

### *T. gondii* CYP activity is derived from Tg-P450

**Figure 5:**
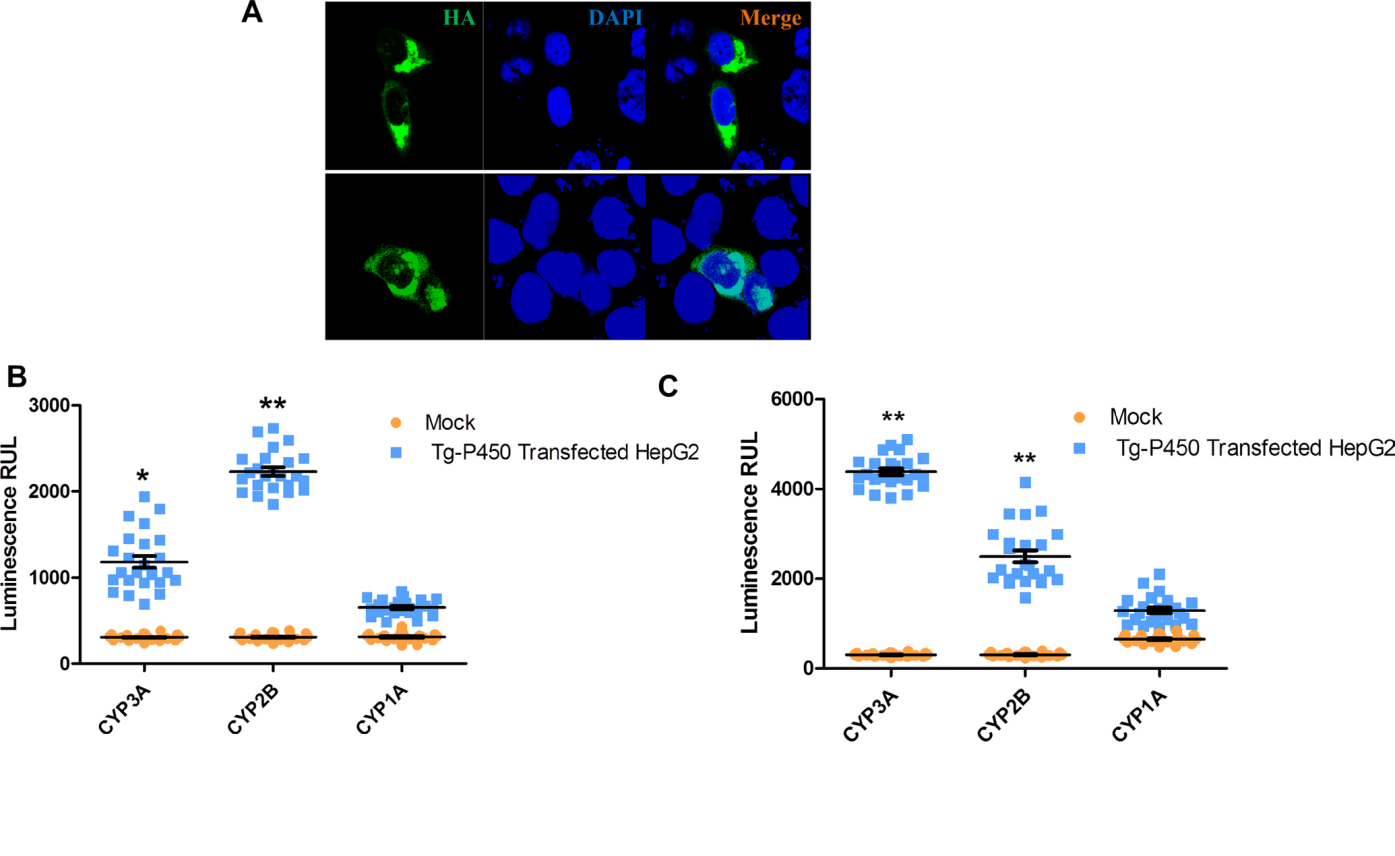
Tg-P450 CYP enzymatic activities in HepG2 cells (**A**) Tg-P450 successfully expressed in HepG2 cells. (**B**) Enzymatic activities of CYP1A, CYP2B and CYP3A in cells transfected with Tg-P450 for 24 h. Note: Comparisons were made with mock cells transfected with the control vector; ^*^P < 0.05, ^**^P < 0.01. (**C**) Enzymatic activities of CYP1A, CYP2B and CYP3A in cells transfected with Tg-P450 and induced with rifampicin (manual recommends a CYP3A inducer) for 24 h. Note: comparisons were made with mock cells transfected with the control vector (also induced with rifampicin); ^*^P < 0.05, ^**^P < 0.01.

To confirm that the *T. gondii* CYP activities were derived from Tg-P450, we examined the effects of Tg-P450 activity in hepatic-derived HepG2 cells, which lack most CYP enzymatic activities [16]. We transfected Tg-P450 into HepG2 cells (Figure 5A) and used P450-Glo™ Assay kits (per the operating manual’s instructions) to determine the enzymatic activity of Tg-P450. The enzymatic activities of P450s in the transfected cells were obviously higher than those in control cells. Tg-P450 was demonstrated to have activity of the mammalian enzymes CYP3A4 and CYP2B. We also examined the activity of CYP1A, which is also responsible for exogenous drug metabolism, revealing that Tg-P450 does not possess this enzyme activity (Figure 5B). Next, we treated transfected cells with the recommended CYP3A inducible agent rifampicin, which increased the activity of CYP3A but not of CYP2B and CYP1A (Figure 5C). The genes *CYP2B* and *CYP3A* encode monooxygenases, *which catalyze* most reactions involved in drug metabolism. Thus, Tg-P450 provides *T. gondii* the ability to resist environments unsuitable for survival.

### Parasites overexpressing Tg-P450 have stronger survival ability than Tg-P450 knockout parasites under drug pressure

**Figure 6:**
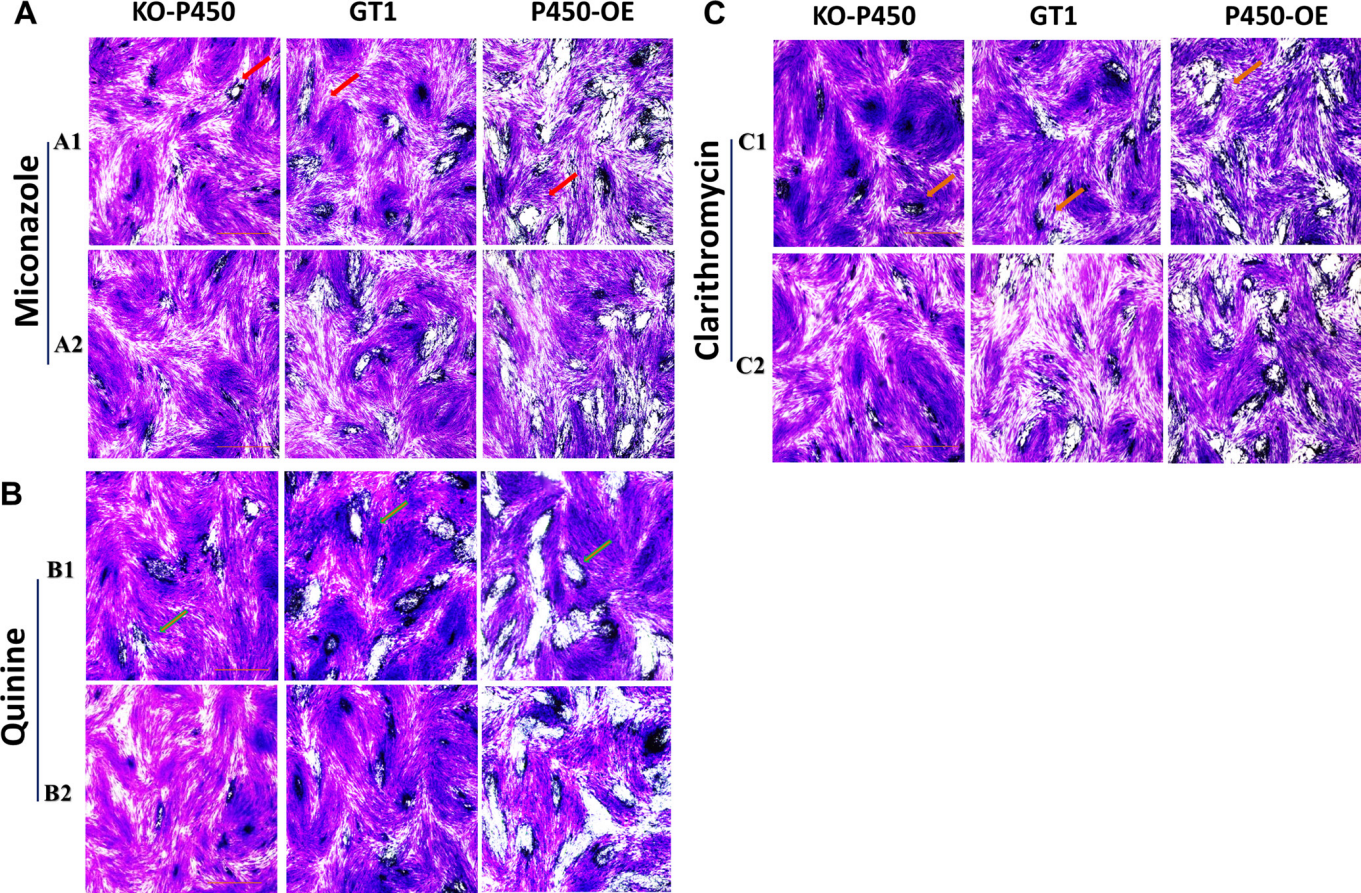
Tg-P450 can render *T. gondii* resistant to exogenous substances (**A**) Effects of miconazole on the intracellular replication of *T. gondii* KO-Tg-P450, Tg-P450 OE and GT1 strain tachyzoites. A1 and A2 were treated with miconazole at concentrations of 10^−2^ μM and 10^−3^ μM for six days. The red arrows point to plaques formed by *Toxoplasma* proliferation (Giemsa staining). (**B**) Effects of quinine on the proliferation of the three strains. B1 and B2 were treated with quinine at concentrations of 6 μM and 0.6 μM. (**C**) Effects of clarithromycin on the proliferation of the three strains. C1 and C2 were treated with clarithromycin at concentrations of 2 × 10^−2^ μM and 2 × 10^−3^ μM.

Plaque assays are often used to assess a multi-step process involving invasion, several rounds of replication and egress. A larger plaque area indicates that *Toxoplasma* is more viable in this environment. To further verify whether Tg-P450 in parasites has anti-drug effects *in* vitro, 500 tachyzoites of the Tg-P450 OE (GT1), KO-Tg-P450 (GT1), and GT1 strains were evaluated in the plaque assay. As shown in Figure 6A, after treatment with miconazole, the Tg-P450 OE strain formed larger plaques and had a stronger vitality than the GT1 strain. Meanwhile, the proliferation of KO-Tg-P450 was the weakest due to lacking the Tg-P450 protein. This trend was maintained even when the miconazole concentration was altered. Subsequently, the same results were obtained when repeating the test with quinine and clarithromycin (Figure 6B–6C), and repeated experiments are shown in Supplementary Figure 1A–1C.

## DISCUSSION

Elucidating the functions of Tg-P450 in this study showed this protein is unnecessary for the survival of *T. gondii* tachyzoites, while P450 overexpression enhanced the pathogenicity of *T. gondii* in mice. Because these results were confusing, we next evaluated whether Tg-P450 confers drug resistance like other organisms, revealing that it possesses CYP3A and CYP2B enzymatic activities but not CYP1A. These enzymatic activities resulted in Tg-P450 OE parasites having high resistance to quinine, mefloquine and clarithromycin.

The Tg-P450 gene exists as only a single copy in the *T. gondii* genome, which is extremely different from humans that have 57 P450 genes. Alternative splicing and genetic variations of human P450 can lead to the production of many more distinct protein species [17], and even in parasites, such as *Haemonchus contortus* (a ruminant nematode), seventy-three partial CYP sequences in 61 supercontig genome assembly databases were identified [18]. The loss or down-regulated expression of CYP family members makes infections with *Schistosoma mansoni* or the fluke *Opisthorchis felineus* lethal [19, 20]. Theoretically, Tg-P450 is essential for parasite survival, as only one CYP450 gene was retained in *T. gondii*. However, the parasites survived even after knocking out the *Tg-450* gene. In *Schistosoma mansoni*, a single P450 protein exists, which is differentially expressed during parasite development in mammalian hosts [19]. Most CYPs were expressed at the highest levels in *H. contortus* in one or more of the four larval stages, but a small number showed higher expression in the egg or in the adult [18]. We performed our studies on only tachyzoite stages, and different developmental stages may require different CYP450 metabolites and/or experience different immunological stresses. Therefore, we cannot absolutely conclude that Tg-P450 is unnecessary for *T. gondii* survival. KCZ is currently recognized as a strong inhibitor of CYP3A, CYP2C and CYP2D, and it is used in many studies [21]. KCZ inhibited the proliferation of *T. gondii* in our study. Nevertheless, knockout of P450 did not affect the survival of parasites. Therefore, the inhibition of *Toxoplasma* survival by KCZ must be due to unknown factors. Among the drugs tested in this study, miconazole (inhibitor of CYP2B6, CYP2C9, CYP2C19, CYP3A4, CYP2A6, and CYP2D6) and clarithromycin (inhibitor of CYP3A4) have the broadest spectrum of inhibitory activity against CYPs [22, 23], and these two drugs have previously exhibited good killing effects on parasites [24, 25]. In our experiments, these two drugs exhibited a perfect killing effect on all of the gene-edited strains, including the Tg-P450 OE strain. In fact, Tg-P450 OE parasites maintained a resistance relatively stronger than that of the GT1 parent strain with a lower drug concentration, but the KO-Tg-P450 parasites effectively lost their resistance. Quinine is a cinchona alkaloid used in the treatment of severe forms of malaria. Formation of 3-hydroxyquinine is catalyzed by CYP3A4 [26]. The concentrations of quinine tested in our study were intended to be approximately equal to the levels required for an inhibitory effect on malaria parasites, but no obvious anti-toxoplasmosis effects were observed, which was similar to previous reports [27]. However, when we knocked out P450 in *T. gondii*, an apparent killing effect was observed. While *Plasmodium* and *T. gondii*, both apicomplexan parasites, have a similar evolutionary relationship, their resistance to quinine differs. Previous reports indicated that malaria parasite resistance may be related to P450 [27], but these claims were eventually denied [28]. Whether the variant functions of P450 in *T. gondii* and *P. falciparum* underlie their differences in resistance needs to be further evaluated.

CYP450s function in electron transport chains wherein electrons are passed from NADPH through a flavoenzyme either directly to the CYP450 heme or indirectly through cytochrome b5 or ferredoxin. In the endoplasmic reticulum, NADPH CYP450 reductase serves as the flavoenzyme [29]. Additional partners of CYP450s in the endoplasmic reticulum include cytochrome b5 and cytochrome b5 reductase [30]. The *T. gondii genome contains one P450* protein, four cytochrome b5s (TGGT1_276110, TGGT1_240770, TGGT1_313580, TGGT1_276990), one cytochrome b5 reductase (TGGT1_262910), and one ferredoxin reductase (TGGT1_215070). As we did not study the metabolic process of the Tg-P450 protein, we have no evidence of Tg-P450 in concert with these proteins. To reveal the function and metabolic process of Tg-P450, a more complex and in-depth study is needed.

Toxoplasmosis remains a challenging disease for people living in endemic areas, and despite many years of drug use, the prevalence of infection remains high. New protein targets for anti-*Toxoplasma* must be identified, and our discoveries described herein may be a new strategy for treatment of this disease. *T. gondii* is typically used as a model protozoon organism because of its easy cultivability and facilitation of gene editing operations, and our results may thus provide evidence for the prevention and control of other protozoon parasites.

## MATERIALS AND METHODS

### Ethics statement

The experiments performed herein were in strict accordance with the recommendations of the Guide for the Care and Use of Laboratory Animals of the Ministry of Science and Technology of China. All experimental procedures were approved by the Institutional Animal Care and Use Committee of China Agricultural University (under the certificate of Beijing Laboratory Animal employee ID: CAU20161210-2). All efforts were made to minimize the pain and suffering of mice during the procedures.

### Parasites and cell culture

Human foreskin fibroblast (HFF), liver hepatocellular carcinoma (HepG2) and African green monkey kidney (Vero) cells were obtained from the Cell Bank of the Chinese Academy of Sciences (Shanghai, China), and cultured in complete Dulbecco’s Modified Eagle’s Medium (DMEM, Macgene, China) as described in previous reports [31]. *T. gondii* tachyzoites (GT1, Pru strains were obtained from Professor Xingquan Zhu, Chinese Academy of Agricultural Sciences) were maintained *in vitro* by serial passage on confluent Vero monolayers in DMEM containing 25 mM glucose and 4 mM glutamine supplemented with 10% fetal bovine serum (FBS, Gibco, USA) and incubated at 37°C with 5% CO_2_ in a humidified incubator. The medium was changed 6 hours after inoculation.

### Mice and experimental infections

Six-week-old BALB/c mice (Peking University Health Science Center, China) were raised with sufficient water and feed, and the light–dark cycle was set at 14 h light:10 h dark. *T. gondii* tachyzoites used to infect mice in all the experiments were suspended in phosphate buffered saline (PBS) and injected intraperitoneally using a 0.25-gauge needle.

### Plaque assay

Plaque assays were performed on HFF cells in 6-well tissue culture plates (Corning Costar, USA). Briefly, 500 tachyzoites per well were seeded onto confluent monolayers, and infected cells were maintained in fresh serum-free medium and incubated without disturbance at 37°C in 5% CO_2_ for 7 days. To stain the monolayers, media was aspirated, and disassociated parasites were removed using PBS. The cell monolayers were then fixed for 10 minutes in PBS containing 4% formaldehyde, stained with crystal violet solution (12.5 g of crystal violet was dissolved in 125 mL of ethanol and mixed with 500 mL of 1% ammonium oxalate in water) at room temperature for 10 minutes. They were then washed with deionized water, air-dried, and visualized by microscopy using image acquisition and plaque area measurements as previously reports described [32].

### Invasion assay

Approximately 1 × 10^4^ tachyzoites were inoculated onto confluent HFF cells in 12-well plates. Invasion was allowed to take place for 30 min at 37°C before replacement of the inoculation medium with fresh medium, and cells were incubated for the following 24 hours. Thereafter, cells were fixed in PBS with 4% formaldehyde, and an indirect immunofluorescence assay (IFA) was performed using a rabbit anti-GAP45 polyclonal antibody (prepared by our laboratory) for staining. In each of three wells, five random fields were captured, yielding 15 total images for each condition. This was repeated for three separate experiments, totaling 45 images analyzed for each condition. Using ImagePro Plus 6.0 software, for each image, the ratio of cells that had been invaded by *T. gondii* (green, FITC labeled) to non-invaded cells was calculated.

### Generation of Tg-P450 knockout and overexpression strains

To generate clean knockouts of specific genes, we used a previously described double sgRNA strategy to create separate double strand breaks at the 5′ and 3′ ends of P450. A CRISPR/CAS9-P450 specific sgRNA sequence (GACGCAAGAGAGAATTGAAG) was designed (E-CRISP, http://www.e-crisp.org/E-CRISP) and used for targeted disruption of the Tg-P450 gene. *P450*-specific sgRNA was cloned into the CRISPR/CAS9 backbone plasmid for *T. gondii* (kindly offered by Bang Shen [33]). In addition, to generate a plasmid for inserting DHFR (pyrimethamine resistance gene) into the Tg-P450 gene, upstream (800 bp) and downstream (750 bp) regions outside the Tg-P450 coding region were used to surround the *DHFR* (primers are listed in Supplementary Table 2).These two plasmids were co-transfected into GT1, and parasites were selected using pyrimethamine.

The complete coding sequence of Tg-P450 was amplified and inserted into a modified pDMG plasmid in which GFP was replaced with HA, and the vector pDMG- Tg-P450-HA was electroporated into GT1 and Pru for Tg-P450 overexpression. The transgenic parasites were selected with pyrimethamine pressure.

### Intracellular parasite replication assay

First, 500 freshly isolated parasites were inoculated on HFF cells in 12-well plates (Corning Costar, USA). After 30 min, the extracellular parasites were removed by washing 3-5 times with PBS. After incubation for 24 h, the infected cells were fixed with 4% formaldehyde, and an IFA was performed using a rabbit anti-GAP45 polyclonal antibody for staining. For these experiments, 100 parasites per vacuole were evaluated, and the results are shown as the mean ± standard deviation.

### Virulence assay in mice

The virulence assay was performed as described previously [34]. For this assay, GT1 (Type I strain) or Pru (Type II strain) tachyzoites were intraperitoneally injected into each mouse, and all infected mice were monitored every 12 h for clinical signs and survival. We terminated the statistics when there were no survivors (type I strain) or after 40 days (type II strain).

### Cloning the Tg-P450 gene and transfection into HepG2 cells

Based on the *T. gondii* protein-coding gene sequence (ToxoDB, TGGT1_315770, TGME49_315770), the primers 5′-ATGTCGGAACTTAGTACGCCTTCGG-3′ forward and 5′-CGCCCGAGGTTTGAAACGAAGCAT-3′ reverse were designed to amplify the full-length Tg-P450 coding sequence. Briefly, total RNA from *T. gondii* tachyzoites was extracted using TRIzol^®^ Reagent (Invitrogen, USA), and first-strand cDNA was synthesized using the above primers with a SuperScript^®^ One-Step RT-PCR System and Platinum^®^ Taq DNA Polymerase (Invitrogen, USA). The PCR fragment containing the Tg-P450 coding sequence was inserted into the pcDNA3.1(+) expression vector to produce the plasmid Tg-P450-pcDNA3.1(+).

### CYP activity test

To measure enzymatic activity, HepG2 cells were transfected with Tg-P450-pcDNA3.1 using Lipofectamine Plus Reagent (Invitrogen, USA); control (mock) cells were also transfected with the empty vector. Transfected cells were incubated at 37°C for 24 h at 5% CO_2_. The CYP1A, CYP2B6, and CYP3A4 enzymatic activities were measured using P450-Glo Assays (Promega, USA) following the manufacturer’s instructions, and reconstitution buffer was added to the Luciferin Detection Reagent.

### In situ visualization assay

Briefly, normal culture medium was replaced with medium containing 1 μM pentoxyresorufin (PR, Anaspect, USA) and benzoxyresorufin (BR, Anaspect, USA). HepG2 cells infected with tachyzoites overexpressing the P450 gene were incubated in 24-well plates at 37°C and 5% CO_2_ for 24 h. Next, samples were washed gently three times with PBS, fixed with 4% formaldehyde (Sigma-Aldrich, USA) for 30 min and mounted on a microscope slide for examination. Tachyzoites in which the P450 gene was knocked out served as the negative control.

### Statistical analysis

Statistical significance between groups was evaluated by two-tailed unpaired Student’s *t*-tests using GraphPad Prism 5 (San Diego, CA, USA). Statistical data are presented as the mean value ± standard error of the mean (SEM). *P* < 0.05 and *P* < 0.01 were considered statistically significant and very significant, respectively.

## SUPPLEMENTARY FIGURE AND TABLES


