# Supplementary material for: Functional characterization of a unique cytochrome P450 in Toxoplasma gondii
Source: Oncotarget. 2017 Dec 6;8(70):115079–88. doi: 10.18632/oncotarget.23023 (PMC5777755; doi:10.18632/oncotarget.23023)
Supplement: Supplementary file 1 [file oncotarget-08-115079-s001.pdf]

Functional characterization of a unique cytochrome P450 in *Toxoplasma gondii*

SUPPLEMENTARY MATERIALS

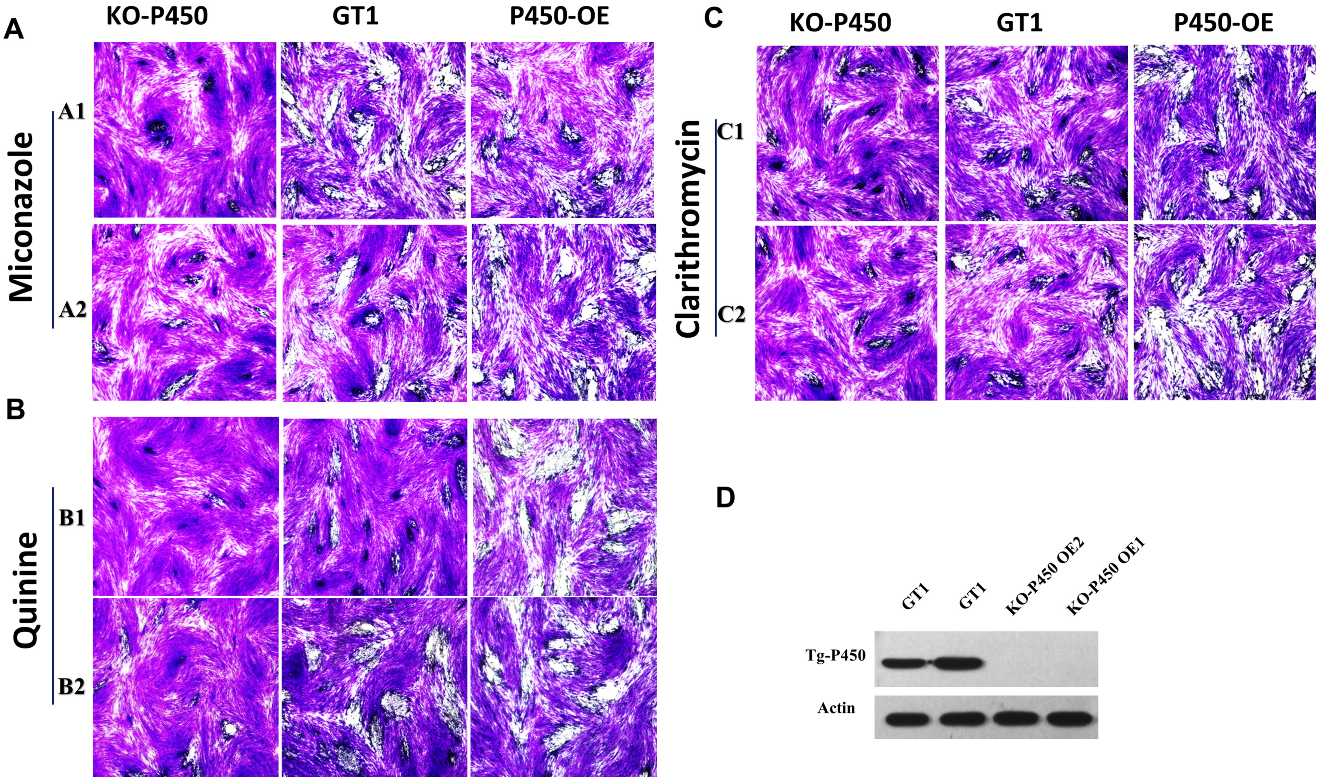

Supplementary Figure 1: Repeated experiments demonstrating that Tg-P450 can render T. gondii resistant to exogenous substance. (**A**) Effects of miconazole on the intracellular replication of T. *gondii* KO-Tg-P450, Tg-P450 OE and GT1 strain tachyzoites. A1 and A2 were treated with miconazole at concentrations of 10–2 µM and 10-3 µM for six days. The red arrows point to plaques formed by *Toxoplasma proliferation* (Giemsa staining). (**B**) Effects of quinine on the proliferation of the three strains. B1 and B2 were treated with quinine at concentrations of 6 µM and 0.6 µM. (**C**) Effect of clarithromycin on the proliferation of the three strains. C1 and C2 were treated with clarithromycin at concentrations of 2×10^{-2} µM and 2×10^{-3} µM. (**D**) Identification of Tg-P450 knockout strains by Western blot.

Supplementary Table 1: KCZ inhibits the proliferation of *T. gondii*

	0.2 mol/L		0.2×10^{-2} mol/L		0.2×10^{-4} mol/L		Control-GT1	
	**Mean**	**SD**	**Mean**	**SD**	**Mean**	**SD**	**Mean**	**SD**
**2**	99	1	11	3.605551	8	2	0.333333	0.57735
**4**	1	1	63.66667	8.736896	43.33333	3.785939	39	6.082763
**8**	0	0	25	4.582576	46	2.645751	51.33333	4.041452
**16**	0	0	0.333333	0.57735	2.333333	2.309401	9.333333	2.309401

Note: Columns indicate numbers of tachyzoites in each parasitophorous vacuole, and rows indicate different concentrations of KCZ used to treat *T. gondii.*

Supplementary Table 2: Primers to generate a plasmid with pyrimethamine-resistant

Primers	Sequence
P450-5′ upstream flank	F: TTAAGCAAAAGCGGCGTGTTG<br>R: GTTGAGGACAGGGTTTAGAAT
P450-3′ downstream flank	F: GGAAAGAAAGGAATCTAGAAGAC<br>R: GTGGCGCAGCAGGCAGTTTGCC
